# Supplementary figures and images for: CryoEM structure and Alphafold molecular modelling of a novel molluscan hemocyanin
Source: PLoS One. 2023 Jun 22;18(6):e0287294. doi: 10.1371/journal.pone.0287294 (PMC10286996; doi:10.1371/journal.pone.0287294)

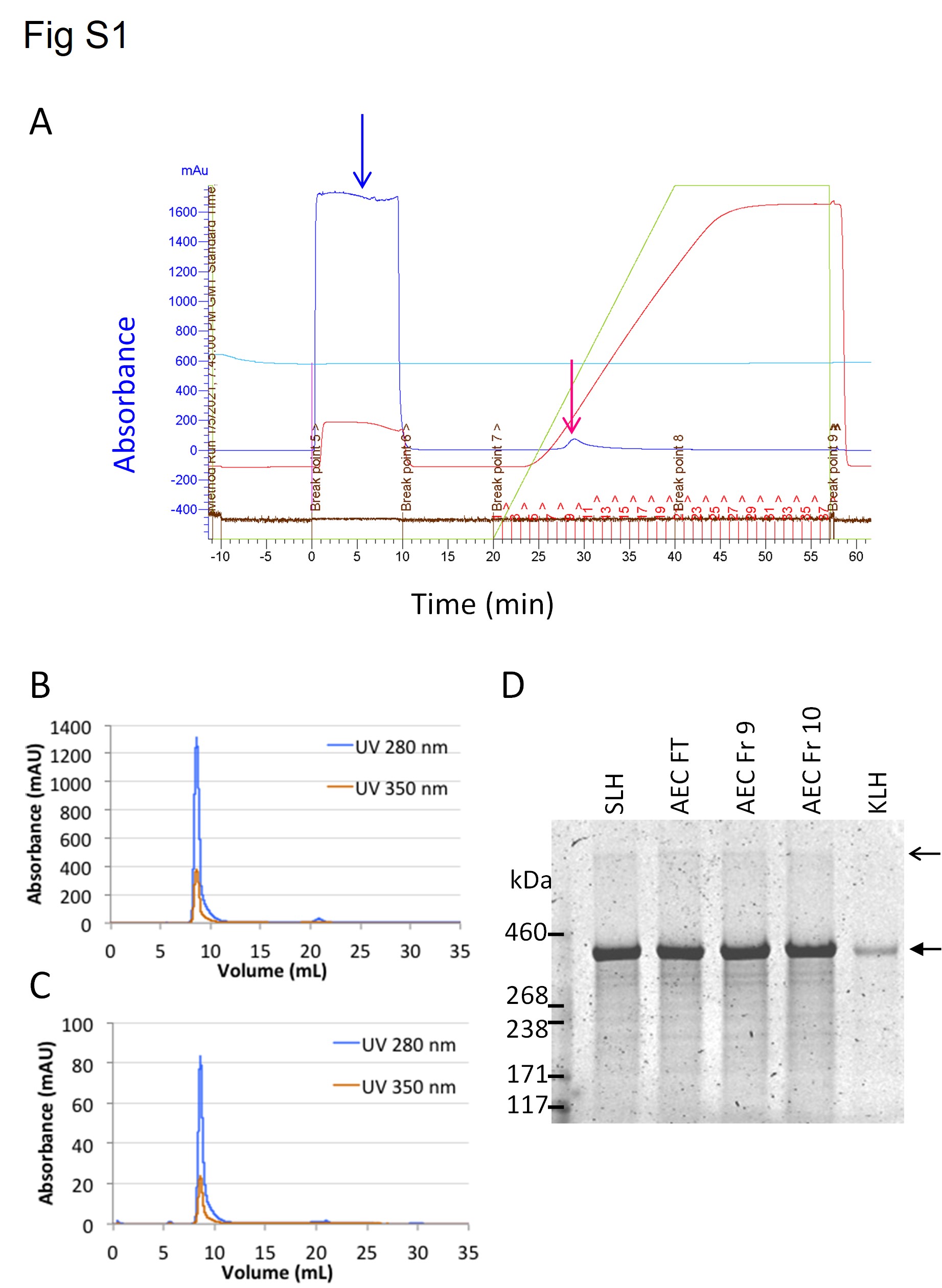

Supplement: S1 Fig — A. Separation of SLH isoforms by Anion Exchange Chromatography (AEC) using HiTrap Q FF 1ml column. Blue trace shows 280 nm protein signal. ~97.5 mg sample load (10 mL loop), Phase 2 buffer (pH 7.4) eluted with gradient of 1M NaCl Phase 2 buffer (green trace showing % of elution buffer). Flow-through peak (blue arrow) plateauing due to the amount of protein loaded and the flow speed of the column (1 ml/min). Eluted minor peak (fractions 9–10) shown by a red arrow. B-C. Gel filtration (Superose® 6 10/300 GL column) traces of flow-through (B) and fraction 9–10 (C) showing 280 nm protein signal (blue) and 350 nm copper-bound signal (orange). D: SDS-PAGE of Size comparison of AEC Flow-through (AEC FT) and AE Fraction 9–10 (AEC Fr 9 and AEC Fr 10, respectively) to un-separated SLH (SLH) and KLH. 1 μg of KLH and 2.5 μg SLH samples were loaded. (JPG) [file pone.0287294.s001.jpg]

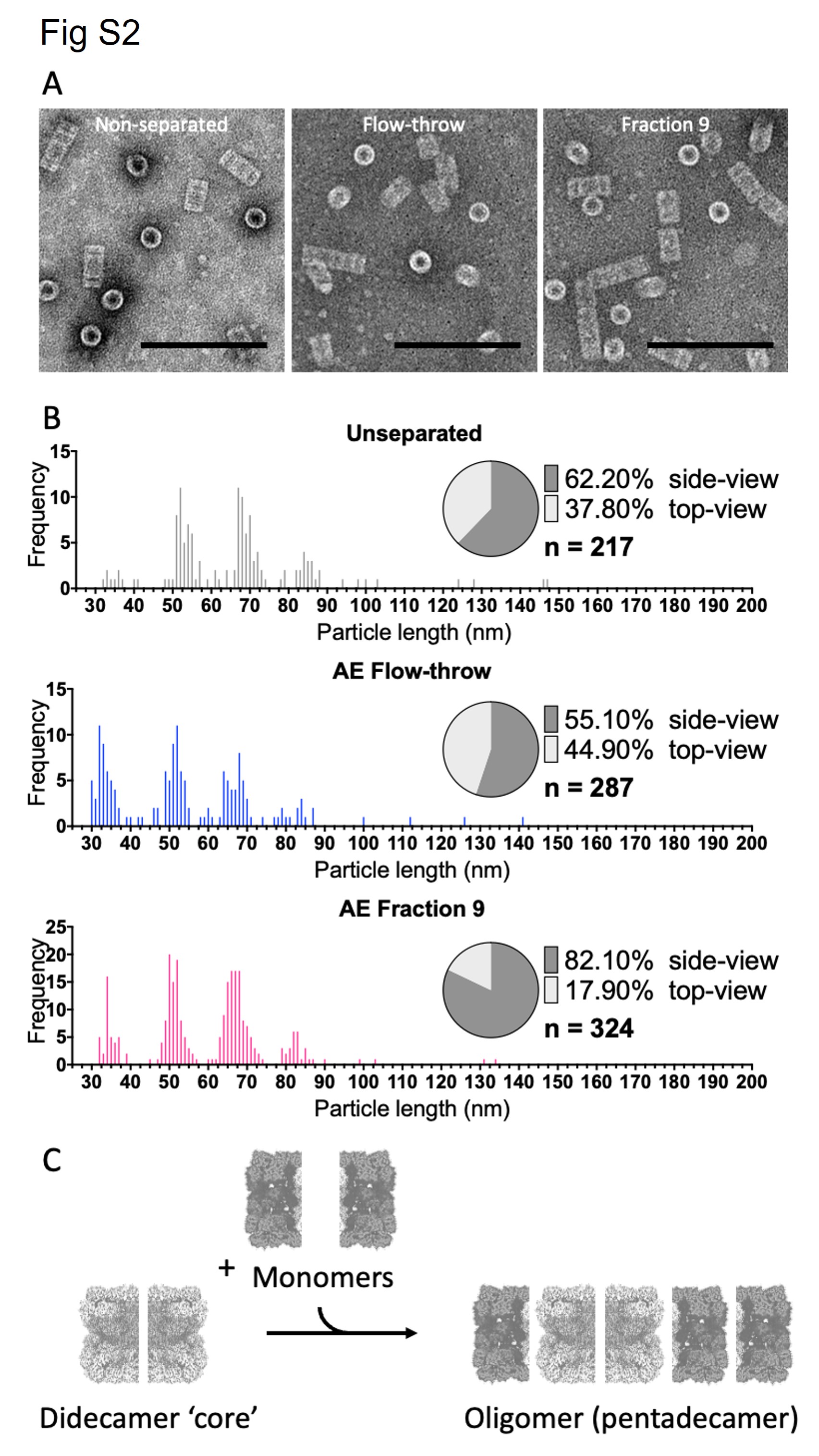

Supplement: S2 Fig — A: Representative transmission electron micrograph of 100 ng/μl flow-through and fraction 9 maintained in Phase 2 buffer negatively stained with UA-Zero. Fraction 9 was ran a total of 3 times through the AE column before the sample was used for grid preparation. Scale bar = 200 nm. B: Histograms of particle length (side-viewed particles only) and pie charts of top:side view ratio of unseparated SLH, flow-through and fraction 9 samples in TEM micrographs. ‘n’ indicates number of measured particles in each sample. C: Cartoon model depicting assembly of higher-order SLH monomers from a ‘core’ didecamer. (JPG) [file pone.0287294.s002.jpg]

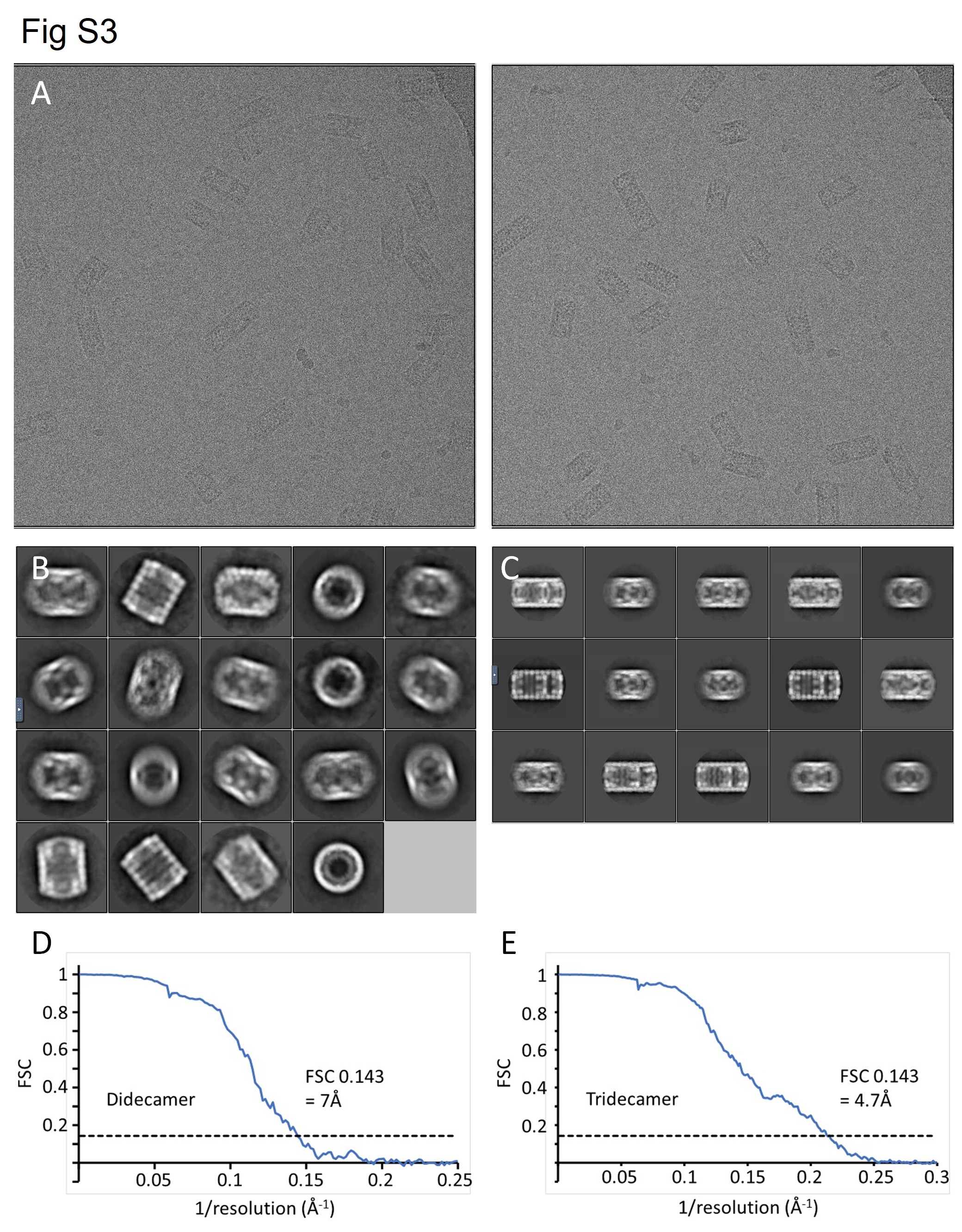

Supplement: S3 Fig — A. Representative micrographs (after motion correction from grids prepared using the chameleon and imaged on the FEI Glacios instrument. Significant particle heterogeneity is observed. B-C. 2D-class averages from the didecamer (B) and tridecamer (C) reconstructions. D-E. FSC plots from the didecamer (D) and tridecamer (E) reconstructions (dashed line represents FSC = 0.143), with final resolutions indicated. (JPG) [file pone.0287294.s003.jpg]

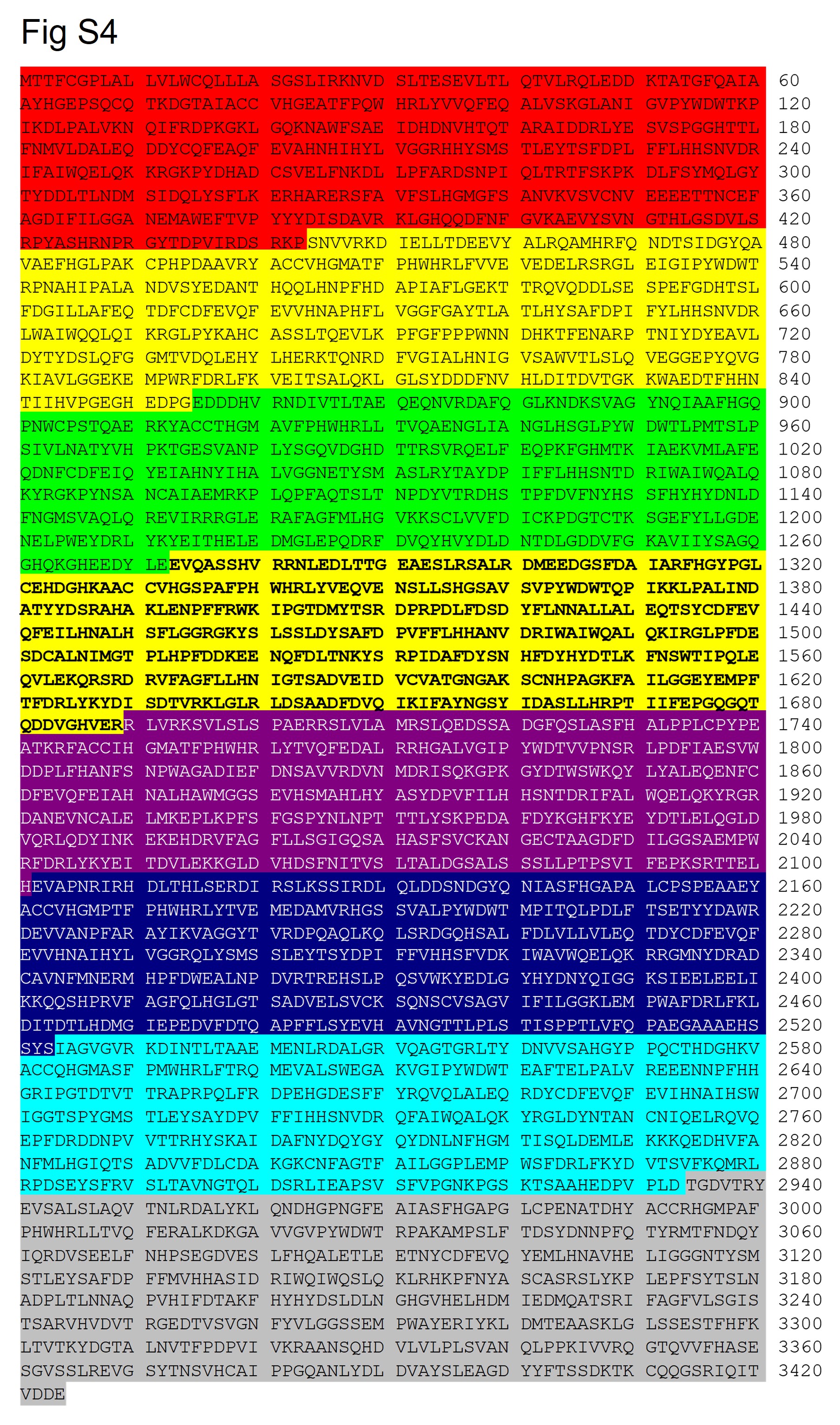

Supplement: S4 Fig — Sequence represents the longest SLH open reading frame (here termed SLH1; derived from publicly deposited transcriptome data) colored by functional unit according to the key in Fig 3 (apart from FU-c (yellow background with bold text) and FU-h (grey background). SLH1 shares 55.5% identity at the amino acid level with KLH1 (Q10583) and 54.9% identity with KLH2 (Q10584). (JPG) [file pone.0287294.s004.jpg]

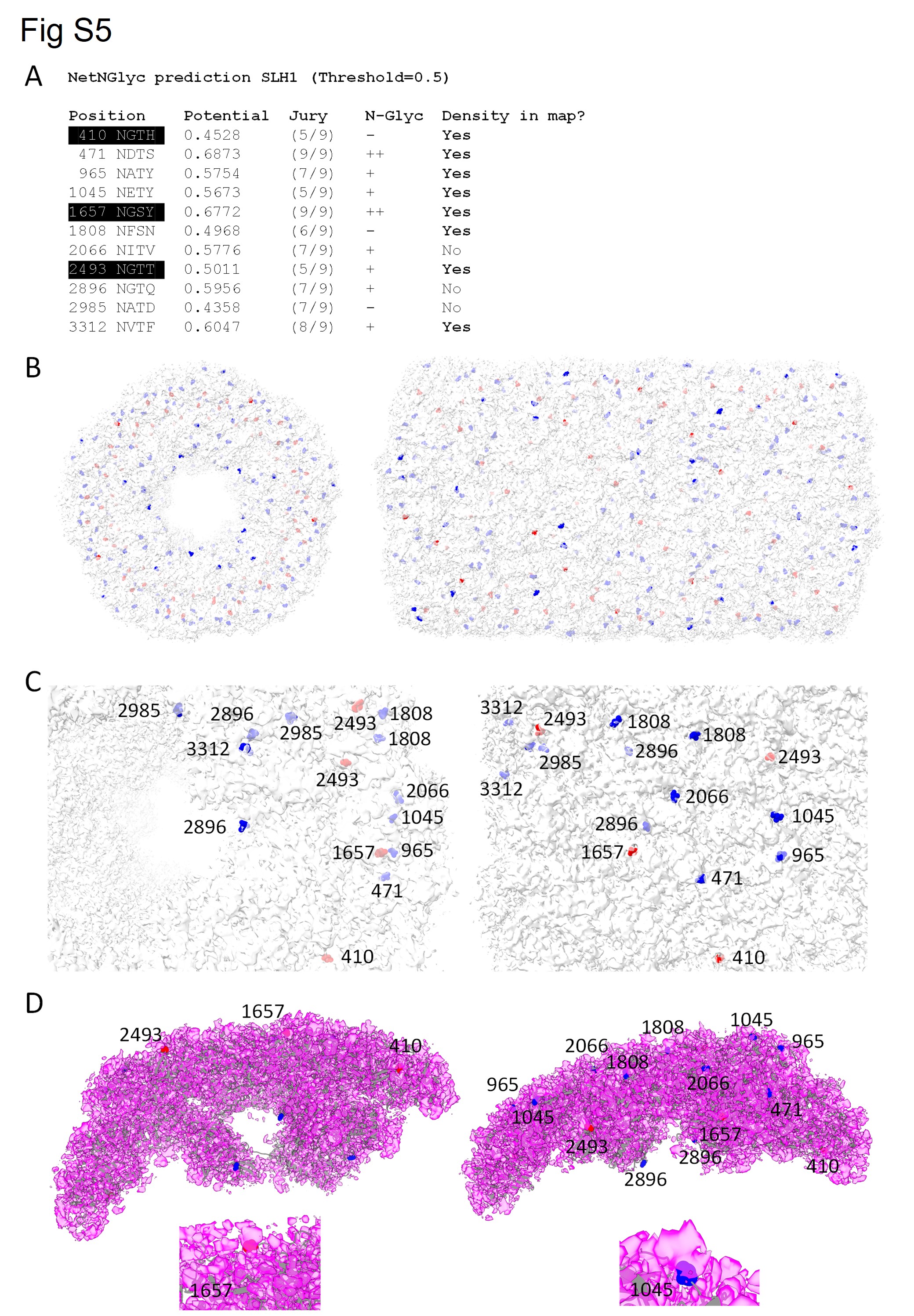

Supplement: S5 Fig — A. NetNGlyc output showing predicted N-glycosylation acceptor sequences for SLH. Sequences conserved between SLH and KLH are highlighted in black. The right-hand column states whether the asparagine side-chain from the alphafold tridecamer model is exposed (No) or buried (Yes) in the cryoEM map; those labelled ‘yes’ may be glycosylated in mature SLH. B. Front and side views of the SLH tridecamer cryoEM structure (white) fitted to the molecular model with conserved (red) and non-conserved (blue) asparagines from potential N-glycosylation sequences shown as sheres. C. Close-up front and side views of the tridecamer structures with asparagine residues labelled; for some, the Asn sidechain is buried within the cryoEM volume (pale red or pale blue), whereas for others the sidechain appears exposed. D. Views of the didecamer cryoEM structure (pink) with the SLH molecular model (grey ribbons) fitted, with potentially glycosylated Asn residues shown as spheres. Close-up views showing the additional density surrounding Asn-1657 and Asn-1045 are also shown. (JPG) [file pone.0287294.s005.jpg]
