# Supplementary material for: CryoEM structure and Alphafold molecular modelling of a novel molluscan hemocyanin
Source: PLoS One. 2023 Jun 22;18(6):e0287294. doi: 10.1371/journal.pone.0287294 (PMC10286996; doi:10.1371/journal.pone.0287294)
Supplement: S1 Table — (PDF) [file pone.0287294.s006.pdf]

Table S1

**Model**	**plDDT**
**1**	*90.48*
2	*89.79*
3	*89.30*
4	*87.39*
5	*88.19*
6	*90.01*
