# Supplementary material for: CryoEM structure and Alphafold molecular modelling of a novel molluscan hemocyanin
Source: PLoS One. 2023 Jun 22;18(6):e0287294. doi: 10.1371/journal.pone.0287294 (PMC10286996; doi:10.1371/journal.pone.0287294)
Supplement: S1 Raw images — (PDF) [file pone.0287294.s007.pdf]

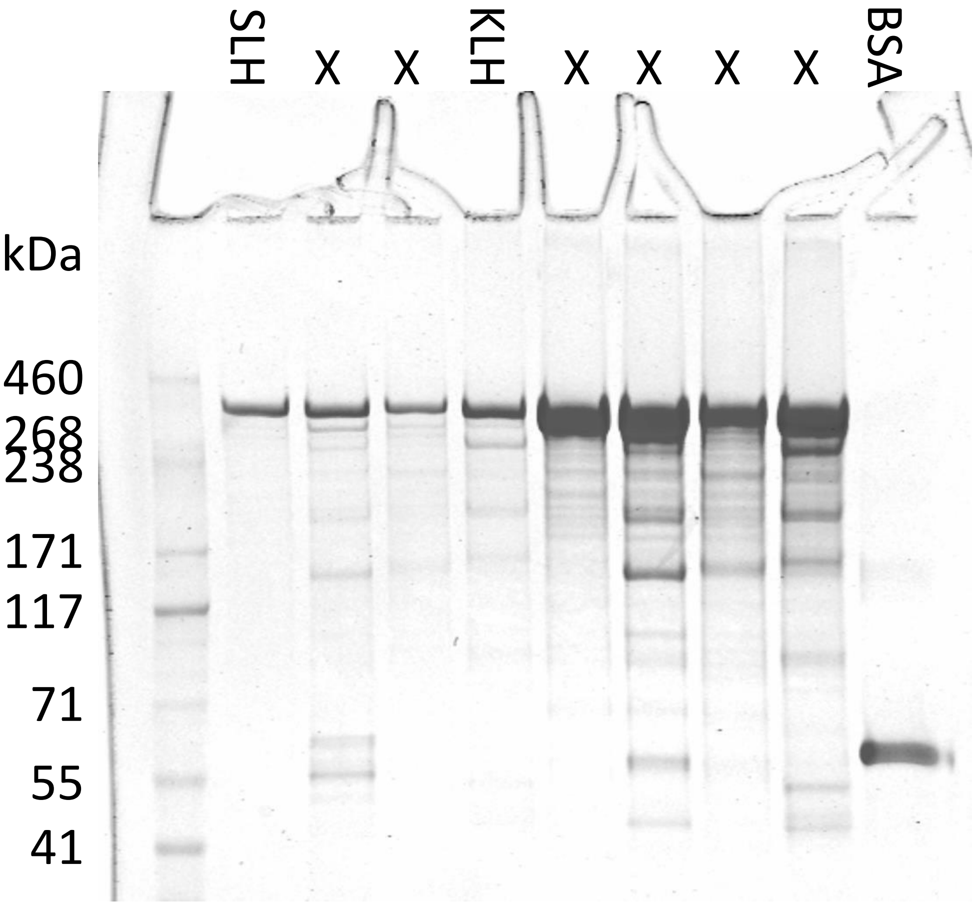


Raw uncropped gel image for Fig 1A.
Gel stain: Instant Blue
Image capture: LiCor Odyssey CLx

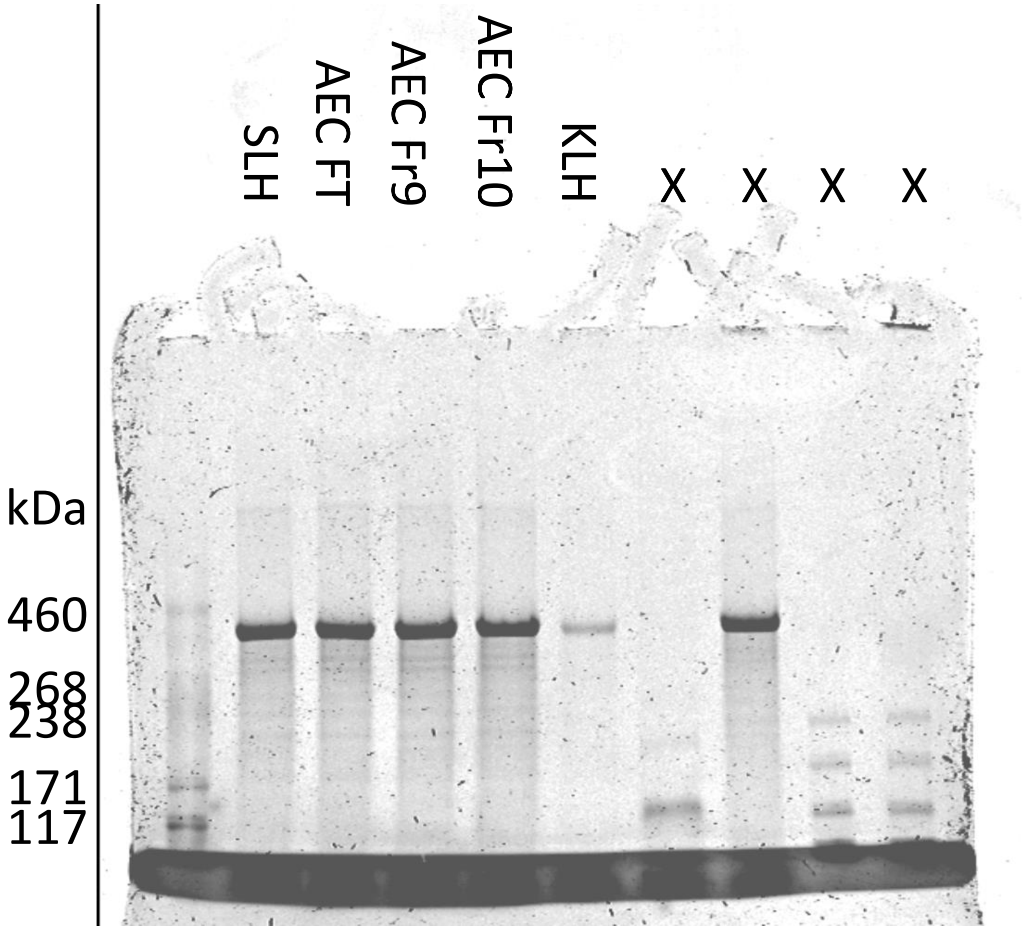


Raw uncropped gel image for Fig S1D.
Gel stain: Instant Blue
Image capture: LiCor Odyssey CLx
