# Supplementary material for: CryoEM structure and Alphafold molecular modelling of a novel molluscan hemocyanin
Source: PLoS One. 2023 Jun 22;18(6):e0287294. doi: 10.1371/journal.pone.0287294 (PMC10286996; doi:10.1371/journal.pone.0287294)
Supplement: Attachment — Submitted filename: Response to reviewers.docx [file pone.0287294.s008.docx]

**PONE-D-23-02379**

**CryoEM structure and Alphafold molecular modelling of a novel molluscan hemocyanin**

**Response to Reviewers**

Please include the following items when submitting your revised manuscript:

- A rebuttal letter that responds to each point raised by the academic editor and reviewer(s). You should upload this letter as a separate file labeled 'Response to Reviewers'.
- A marked-up copy of your manuscript that highlights changes made to the original version. You should upload this as a separate file labeled 'Revised Manuscript with Track Changes'.
- An unmarked version of your revised paper without tracked changes. You should upload this as a separate file labeled 'Manuscript'.

If you would like to make changes to your financial disclosure, please include your updated statement in your cover letter. Guidelines for resubmitting your figure files are available below the reviewer comments at the end of this letter.

**Journal Requirements**

1. Please ensure that your manuscript meets PLOS ONE's style requirements, including those for file naming. The PLOS ONE style templates can be found at: <https://journals.plos.org/plosone/s/file?id=wjVg/PLOSOne_formatting_sample_main_body.pdf> and <https://journals.plos.org/plosone/s/file?id=ba62/PLOSOne_formatting_sample_title_authors_affiliations.pdf>

This has been checked and we have made every attempt to follow the PLOS ONE style requirements.

2. We note that the grant information you provided in the ‘Funding Information’ and ‘Financial Disclosure’ sections do not match. When you resubmit, please ensure that you provide the correct grant numbers for the awards you received for your study in the ‘Funding Information’ section.

This has been corrected.

3. In your Data Availability statement, you have not specified where the minimal data set underlying the results described in your manuscript can be found. PLOS defines a study's minimal data set as the underlying data used to reach the conclusions drawn in the manuscript and any additional data required to replicate the reported study findings in their entirety. All PLOS journals require that the minimal data set be made fully available. For more information about our data policy, please see <http://journals.plos.org/plosone/s/data-availability>. Upon re-submitting your revised manuscript, please upload your study’s minimal underlying data set as either Supporting Information files or to a stable, public repository and include the relevant URLs, DOIs, or accession numbers within your revised cover letter. For a list of acceptable repositories, please see http://journals.plos.org/plosone/s/data-availability#loc-recommended-repositories. Any potentially identifying patient information must be fully anonymized. **Important:** If there are ethical or legal restrictions to sharing your data publicly, please explain these restrictions in detail. Please see our guidelines for more information on what we consider unacceptable restrictions to publicly sharing data: http://journals.plos.org/plosone/s/data-availability#loc-unacceptable-data-access-restrictions. Note that it is not acceptable for the authors to be the sole named individuals responsible for ensuring data access. We will update your Data Availability statement to reflect the information you provide in your cover letter.

The raw cryoEM image data has been uploaded to EMPIAR (EMPIAR-11554) and this has been indicated in the manuscript.

4. We note that you have stated that you will provide repository information for your data at acceptance. Should your manuscript be accepted for publication, we will hold it until you provide the relevant accession numbers or DOIs necessary to access your data. If you wish to make changes to your Data Availability statement, please describe these changes in your cover letter and we will update your Data Availability statement to reflect the information you provide.

The repository accession numbers are in the manuscript, and if our manuscript is accepted we will ensure that these accessions are publicly available.

5. PLOS ONE now requires that authors provide the original uncropped and unadjusted images underlying all blot or gel results reported in a submission’s figures or Supporting Information files. This policy and the journal’s other requirements for blot/gel reporting and figure preparation are described in detail at https://journals.plos.org/plosone/s/figures#loc-blot-and-gel-reporting-requirements and https://journals.plos.org/plosone/s/figures#loc-preparing-figures-from-image-files. When you submit your revised manuscript, please ensure that your figures adhere fully to these guidelines and provide the original underlying images for all blot or gel data reported in your submission. See the following link for instructions on providing the original image data: https://journals.plos.org/plosone/s/figures#loc-original-images-for-blots-and-gels. In your cover letter, please note whether your blot/gel image data are in Supporting Information or posted at a public data repository, provide the repository URL if relevant, and provide specific details as to which raw blot/gel images, if any, are not available. Email us at plosone@plos.org if you have any questions.

Uncropped raw images for the 2 gel figures are provided in Figure S6.

6. We note that you have included the phrase “data not shown” in your manuscript. Unfortunately, this does not meet our data sharing requirements. PLOS does not permit references to inaccessible data. We require that authors provide all relevant data within the paper, Supporting Information files, or in an acceptable, public repository. Please add a citation to support this phrase or upload the data that corresponds with these findings to a stable repository (such as Figshare or Dryad) and provide and URLs, DOIs, or accession numbers that may be used to access these data. Or, if the data are not a core part of the research being presented in your study, we ask that you remove the phrase that refers to these data.

We have removed these phrases.

7. Please review your reference list to ensure that it is complete and correct. If you have cited papers that have been retracted, please include the rationale for doing so in the manuscript text, or remove these references and replace them with relevant current references. Any changes to the reference list should be mentioned in the rebuttal letter that accompanies your revised manuscript. If you need to cite a retracted article, indicate the article’s retracted status in the References list and also include a citation and full reference for the retraction notice.

To the best of our knowledge this list is correct.

8. While revising your submission, please upload your figure files to the Preflight Analysis and Conversion Engine (PACE) digital diagnostic tool, https://pacev2.apexcovantage.com/. PACE helps ensure that figures meet PLOS requirements. To use PACE, you must first register as a user. Registration is free. Then, login and navigate to the UPLOAD tab, where you will find detailed instructions on how to use the tool. If you encounter any issues or have any questions when using PACE, please email PLOS at figures@plos.org. Please note that Supporting Information files do not need this step.

Figures have been uploaded to PACE for checking – all figures are OK apart from fig 5 which uploaded and looks OK, but PACE states that the file size (when converted to TIFF) is invalid. I do not know how to resolve this, but if it is a problem I can remake the figure to try to reduce file size.

**Reviewer 1**

Authors of this paper describe the cryoEM structure of the didecamer and tridecamer froms of a novel hemocyanin from the SLH, and use Alphafold to build large oligomeric proteins. However, I have some questions:

1. In SI page 4, line 11, Fig S4, some of them are identified by the color of the font itself, while others are identified by background highlighting，please use the same identification method to identidy functional units.

We have used the background highlighting for the functional units to keep this consistent.

2. In paper page 18, line 395-396, "The sequence provided was split into 6 sequences which were a maximum of 800 amino acid in length with a portion overlapping". What is the basis for the truncation except for the maximum limit of Alphafold?

The basis for this truncation was indeed the maximum limit for Alphafold.

**Reviewer 2**

The work of science is highly interesting which elucidates the 3D-structure of slipper limpet hemocyanin, combining cryoEM with Alphafold modelling, and demonstrated how higher-order oligomers can form in hemocyanins of the KLH class. I have some questions which I feel can help in the development of the work.

1. The authors have explained that they can see higher order oligomers in the sample produced. I would like to know which part of the protein helps in this oligomerisation. SEC-MALS studies as well as mutational studies ca explain the oligomerisation formation.

This is a very interesting suggestion. We looked at using SEC-RALS to look at oligomer formation, but we were not able to resolve single peaks for any species in our SEC experiments and so we did not continue with these experiments. From visual inspection of our negative-stain and cryoEM images we always observe the core didecamer (a tail-tail interaction) in any oligomer, and then additional subunits add on in a head-tail interaction. From our fitting of the Alphafold models to the tridecamer structure, we observe a small difference in the subunit interface in the head-tail interaction compared to the tail-tail interaction. It may be that in other hemocyanins that do not show substantial higher-order oligomer formation, this interaction is prevented or disfavoured by glycosylation or simply lack of an interface. Mutational studies are an excellent suggestion to resolve this, but we are working with protein purified from a native source in an organism that is not amenable to cloning for site-directed mutagenesis, so it is not possible for us to address this point. We have included some additional information in the discussion to cover this point (page 12).

2. How important s it for hemocyanin to form oligomer for its functionality? Any in-vitro experiment elucidating the function can help in the understanding of the formation of higher order oligomers.

This is a very interesting point – unfortunately we do not know about the biological relevance of the higher order oligomers, or indeed the oxygen binding or enzyme function of our SLH didecamer, but from comparison with other hemocyanins, we know that oxygen binding is cooperative (so subunit-subunit interactions are critical for this process), and that partial denaturation or proteolysis produces a functional oxidase enzyme with a ‘relaxed’ structure relative to the intact protein. Functional experiments are something that we would very much like to address in future experiments, but we think they are beyond the scope of the structural and modelling experiments that we present in this manuscript. We have included some additional information in the text (pages 12 and 13) to discuss these points.

3. A cartoon model of the formation of oligomer from monomer can help in better understanding of the work.

We agree that this information would be very useful. We do not have any experimental evidence for how a decamer might form from a monomer (we assume a monomer forms a dimer ‘plate’ and then 5 ‘plates assemble to the didecamer). We have included a simple cartoon model describing how decamers may join together to assemble didecamers and higher-order structures (Fig S2C).
